# Accessing Disadvantaged Pregnant Women in Houston, Texas, and Characterizing Biomarkers of Metal Exposure: A Feasibility Study

**DOI:** 10.3390/ijerph14050474

**Published:** 2017-04-29

**Authors:** Kristina W. Whitworth, Inkyu Han, Masoud Afshar, Yuan Mei, Pamela D. Berens, Shreela V. Sharma, Elaine Symanski

**Affiliations:** 1 Department of Epidemiology, Human Genetics, and Environmental Sciences, UTHealth School of Public Health in San Antonio, San Antonio, TX 78229, USA; 2 Southwest Center for Occupational and Environmental Health, UTHealth School of Public Health, Houston, TX 78030, USA; Inkyu.Han@uth.tmc.edu (I.H.); masoud.afshar@uth.tmc.edu (M.A.); Elaine.Symanski@uth.tmc.edu (E.S.); 3 Department of Epidemiology, Human Genetics, and Environmental Sciences, UTHealth School of Public Health, Houston, TX 78030, USA; Yuan.Mei@uth.tmc.edu (Y.M.); Shreela.V.Sharma@uth.tmc.edu (S.V.S.); 4 Department of Obstetrics, Gynecology, and Reproductive Sciences, UTHealth McGovern Medical School, Houston TX 78030, USA; Pamela.D.Berens@uth.tmc.edu

**Keywords:** disadvantaged populations, pregnant women, prenatal, blood metal, biomarker

## Abstract

Communities of color or low socioeconomic status are disproportionately affected by metal exposure given spatial variability of the ambient levels of these contaminants. Despite this, there is little research characterizing metal concentrations in blood among disadvantaged populations in the U.S., especially among pregnant women who are particularly vulnerable and difficult to access. Thus, we conducted a pilot study among disadvantaged pregnant women in Houston, Texas to assess willingness to participate in key activities of an epidemiologic study and characterize exposures to 16 metals. Thirty-one women attending a Medicaid-serving prenatal clinic were included in this pilot study and completed an interviewer-administered questionnaire. We obtained and measured metal compounds in whole blood samples for 22 of these women during third-trimester prenatal visits. Median whole blood concentrations of Ni, As, Cd, and Pb were 27, 1.4, 0.6, and 6.3 µg/L, respectively. Most women were willing to participate in critical aspects of a research study, including wearing a personal air-sampling badge for 2–3 days (87.1%), receiving ultrasounds (83.9%), and providing blood draws (64.5%). Despite the small sample, our results provide evidence of women’s metal exposure and their willingness to participate in future research studies to elucidate exposure pathways and explore related health effects experienced among this population of disadvantaged pregnant women.

## 1. Introduction

Metals are ubiquitous in the environment due to biological and geological cycles, as well as anthropogenic activities, such as smelters or hazardous waste sites [1]. Metals are chemically stable and persistent in the human body and the environment; the primary routes of human exposure to most metals is inhalation or ingestion [2]. Pregnant women and the developing fetus are particularly susceptible to the effects of environmental contaminants, and many metals compounds can cross the placental barrier [3]. There is increasing evidence regarding adverse maternal and child health outcomes related to metals exposures including spontaneous abortion, reduced fetal growth, and neurodevelopmental delays [4,5] 

Often, environmental risks are not uniformly distributed in urban areas and minorities and individuals classified with low socioeconomic status (SES) may be overburdened by environmental metals exposure [6,7,8,9]. Further, Morello-Frosch and Shenassa [10] propose a mechanism through which individual-level (e.g., poor nutrition) and community-level (e.g., neighborhood economic deprivation) psychosocial stressors affect allostatic load and may thus modify effects of environmental exposures and contribute to maternal and child health disparities. Consequently, studies should prioritize ongoing biomonitoring and characterization of metal exposure in at-risk populations, such as pregnant women belonging to overburdened segments of society such as minority and low-income groups. However, these groups have been historically underrepresented in research. A recent review indicates a mistrust, competing demands, and lack of access to information as important barriers of participation in research studies among minorities, including African Americans [11]. 

We conducted a pilot study among disadvantaged pregnant women in Houston, Texas, to assess their willingness (1) to participate in key activities of a future epidemiologic study of maternal and child health and (2) provide a single blood sample to characterize their metal exposures. 

## 2. Materials and Methods

This pilot study was conducted from 2015–2016 and was based upon an ongoing intervention targeting healthy eating, breastfeeding, and physical activity: Healthy Eating Active Living (HEAL). Women in HEAL are recruited from a prenatal health clinic in Houston, TX, which primarily serves patients receiving Medicaid health coverage, which is available to low-income pregnant women. As part of their eligibility for HEAL, women must be either Medicaid-eligible or enrolled in Medicaid. Additional eligibility criteria in our study included aged ≥18 years, 20–33 weeks of gestation, English-speaking, and not intending to change health care providers during pregnancy. Following in-person interviews, HEAL staff referred all women interested in participating in the pilot to our staff. Women who were not referred in-person (e.g., the woman did not show up to their scheduled HEAL interview or did not have time following the HEAL interview) were recruited by phone. Women provided written informed consent, agreed to metal analysis of their routine third trimester blood draw, and completed an interviewer-administered questionnaire about residential history, smoking habits, and willingness to participate in future studies of pregnant women and their babies (demographic information was obtained from HEAL). Women received a $20 gift card for participation. This study received institutional review board approval from the University of Texas Health Science Center at Houston to ensure ethical standards for human research were met and maintained (IRB #HSC-SPH-15-0331).

The phlebotomist placed blood samples in a cooler and study staff transported them within three hours to the laboratory where they were immediately refrigerated and then analyzed within six weeks of collection. A 0.5 mL of homogenized blood sample (equilibrated at room temperature) was transferred using a glass pipette into a trace element-free vessel and spiked with 0.5 mL of internal standards (at 1 ppm) consisting of Scandium (Sc), Rhodium (Rh), Terbium (Tb), and Bismuth (Bi). We acidified the samples with a mixture of ultra-pure grade of trace element-free 2% nitric acid and 0.5% hydrochloric acid to make a final volume of 10 mL (1:20 ratio dilution). This sample treatment was developed by modifying a method used by Zhang et al. [12]. Each tube was centrifuged at 4000 rpm for 15 min using an Eppendorf 5810 (Eppendorf, Hauppauge, NY, USA) and an aliquot from the top was analyzed for metals. 

All samples were placed on an autosampler tray and introduced into Inductively Coupled Plasma Mass Spectrometry (ICP/MS) (ICP/MS 7300ce, Agilent Technologies, Palo Alto, CA, USA). We prepared standards using 2% nitric acid and 0.5% hydrochloric acid (ultra-pure trace element grade) and known quantities of aluminum, vanadium, chromium, manganese, cobalt, nickel, copper, zinc, arsenic, selenium, strontium, silver, cadmium, antimony, barium, and lead in a linear range from 0.1 to 1000 ng/mL. The maximum detected metal concentrations in blood are around 20,000 ng/mL (or µg/L) due to a 1:20 dilution ratio. We determined the limit of detection (LOD) as three times the standard deviation of seven replicate analyses of a blank solution. Blood metal levels were adjusted for blank concentrations and expressed in units of µg/L. Blank-adjusted samples below, the LOD were replaced by the LOD/$\sqrt{2}$. 

For quality control, we performed three tasks: First, we examined the background concentrations of metals in empty vacutainer tubes by adding 10 mL of 18.2 MΩ resistance de-ionized water (Milli-Q water purification system, Millipore, Bedford, MA, USA) to an empty vacutainer tube and followed the same analysis procedure used for blood samples. Through this procedure, we confirmed that all metal concentrations were either not detected or below the LOD. Second, we tested the performance of our method using synthetic blood (Colorado Serum Company, Denver, CO, USA). We added 0.5 mL of synthetic blood into an empty vacutainer and processed the sample as described above. With our analyses, we verified that metal concentrations in synthetic blood were either not detected or below the LOD. Lastly, we prepared two different sets of five vacutainers each containing 0.5 mL of synthetic blood: the first set of five vacutainers were spiked with 10 ppb for each metal and the second set of five vacutainers were spiked with 100 ppb for each metal. We processed all these quality control samples in the same manner as described above and assessed the recovery rates for each metal, which were between 87% and 117%. 

We conducted descriptive analyses of participant characteristics and metal concentrations in blood using SAS version 9.3 (Cary, NC, USA).

## 3. Results

**Figure 1:**
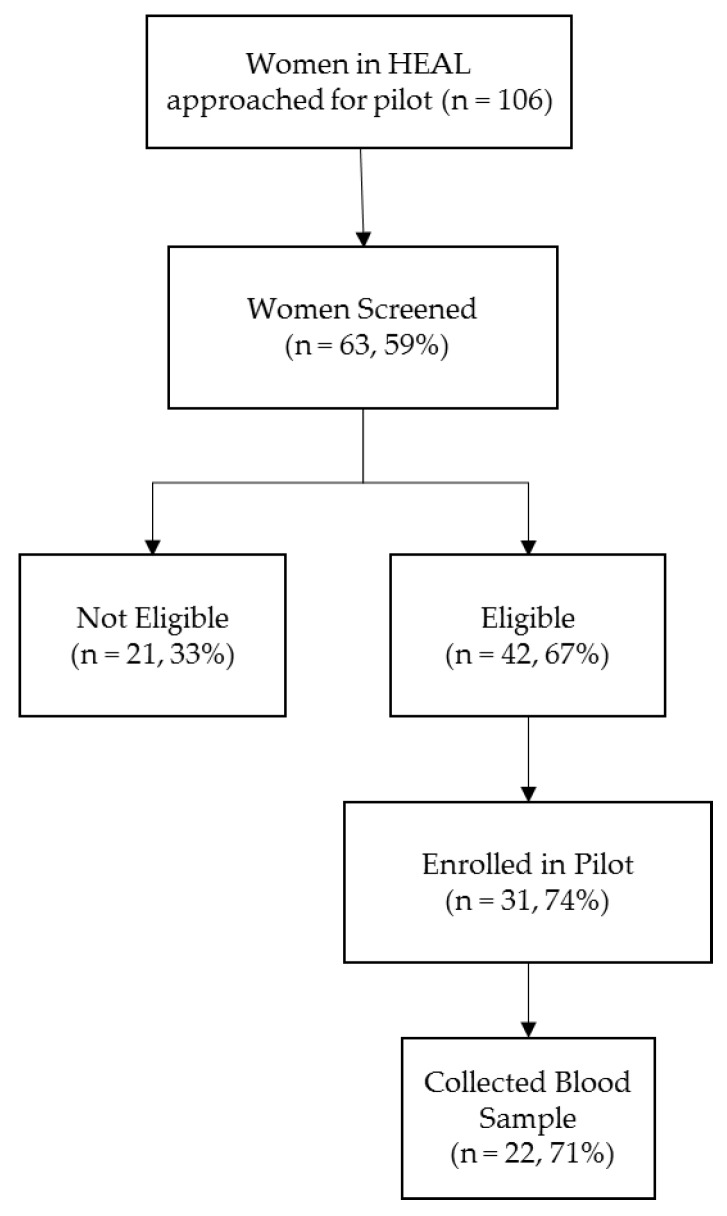
Flow chart of study recruitment.

As part of this pilot study, we approached 106 women from HEAL for participation and 63 of them completed the eligibility screener (Figure 1). The most common reason for not completing the screener was the lack of time (49%). Among women screened, 42 were eligible. Most women were ineligible because they were <20 weeks of gestation (71%). Thirty-one women enrolled in the study: most were recruited in-person (21 out of 22) rather than by phone (10 out of 20). We obtained blood from 22 women; three women did not have blood drawn in the clinic, two gave birth prior to the blood draw, one woman was lost at follow-up, and three women declined the blood draw.

Women’s mean age at enrollment was 29 years (±4.9) and mean gestational age was 25 weeks (±4.1) (Table 1). The majority of women were black, non-Hispanic (41.9%), or Hispanic (29.0%). Most women had never married (35.5%) and had a high school education or less (38.7%). Nearly half (45.2%) reported an annual household income of <$15,000. Few women reported smoking (*n* = 3; 9.7%) or second-hand smoking in the home (*n* = 3; 9.7%) during pregnancy. Less than half (35.5%) worked outside the home and only six (19.4%) moved during pregnancy. Of the women who moved during pregnancy, five women reported moving once, and one woman reported moving twice (data not shown). The distribution of demographic characteristics among the 22 women with a blood draw was similar to all women in the pilot study.

When asked about their willingness to take part in a future study, most women stated they would allow researchers to obtain outdoor residential air samples (57.1%) or wear a personal air-sampling badge for several days (87.1%) (Table 2). They also indicated a willingness to have additional ultrasounds (83.9%) and blood draws (64.5%). Fewer women were willing to provide a cord blood sample (51.6%) or access to children’s medical records (45.2%) and birth certificates (41.9%). Approximately one-third of women were unsure whether they would participate in each of these three aspects of a future study.

**Figure 2:**
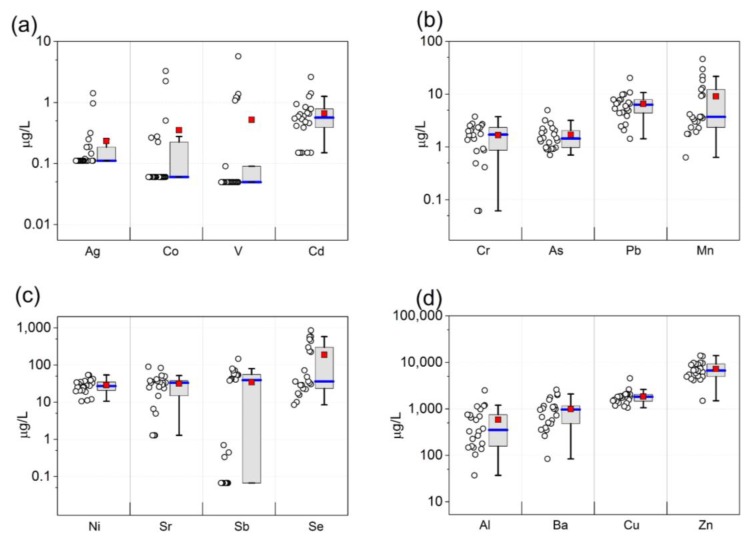
Distribution of metals in the whole blood of 22 pregnant women attending a primarily Medicaid-serving prenatal clinic in Houston, Texas, 2015–2016. Each data point represents an individual metal concentration value in blood. Red squares represent mean concentrations and blue lines represent median concentrations: (**a**) Ag, Co, V, and Cd concentrations in whole blood; (**b**) Cr, As, Pb, and Mn concentrations in whole blood; (**c**) Ni, Sr, Sb, and Se concentrations in whole blood; (**d**) Al, Ba, Cu, and Zn concentrations in whole blood.

Except for V, Co, and Ag, we detected each of the metals in at least two-thirds of samples (Table 3). The highest median concentrations were detected for Zn, Cu, Al, and Ba (Table 3, Figure 2). For most metals, there was little change in these results when we excluded three women reporting smoking, including Ni (GM = 27 µg/L; median = 28 µg/L), As (GM = 1.5 µg/L; median = 1.5 µg/L), Cd (GM = 0.42 µg/L; median = 0.55 µg/L), and Pb (GM = 5.2 µg/L; median = 6.0 µg/L). Concentrations of Al (GM = 432 µg/L; median = 577 µg/L) and Ba (GM = 881 µg/L; median = 1006 µg/L) increased when smokers were excluded.

## 4. Discussion

We successfully implemented a pilot study of disadvantaged pregnant women in Houston, TX, which included completing an in-person interviewer-administered questionnaire and providing a blood sample to characterize metal exposures. Our results suggest that these pregnant women are exposed to several potentially toxic metals, including Cd, Pb, Ni, and As. Our findings also have direct implications for the design and conduct of future epidemiologic studies focused on maternal and child health among this vulnerable and understudied population. 

Though our study did not set out to test different recruitment strategies and is limited by a small sample size, we recruited more women via in-person contact than phone contact. We provide evidence of women’s willingness to allow for outdoor air monitoring at their homes, provide biological samples, and undergo additional ultrasound testing during pregnancy. Though fewer women indicated willingness to participate in other aspects of a research study of maternal and child health (e.g., provide a cord blood sample, provide access to a child’s birth certificate, and provide access to medical records), the majority of women did not respond negatively to these questions. Importantly, one-third or more of the women said they did not know whether they would participate in these aspects of a future study, indicating potential lack of knowledge regarding exactly what they may be asked to do. In our pilot study, the interviewer simply asked the woman if they would be willing to participate in these activities with no additional explanation provided. We think these results should be viewed as encouraging rather than discouraging, though they reveal the potential necessity to pilot test questionnaires to ensure that explanations of study protocols are clear and comprehensive when recruiting this population in future studies. This is in line with previous studies that indicated having safety assurances may facilitate research participation, particularly among African Americans [11,13].

While there are investigations of metal exposures among pregnant women outside of the U.S. [5,14,15], we restrict our comparisons of metal concentrations in blood to other studies of pregnant women in the U.S. or Canada. For Cd, other North American studies report geometric mean (GM) or median blood concentrations ranging from 0.18 µg/L to 0.48 µg/L [16,17,18] or 0.20 µg/L to 0.40 µg/L [19,20,21,22], respectively. A few studies reported GM Cd concentrations specifically among non-smokers that were similar in magnitude to each other (0.18 µg/L [19]; 0.20 µg/L [22]; 0.27 µg/L [16]), but lower than what we observed among non-smokers (GM = 0.42 µg/L). Two previous studies reported GM blood Pb concentrations among pregnant women from North Carolina and from several Canadian regions equal to 8.9 µg/L [17] and 6.0 µg/L [18], respectively; each of these studies reported higher blood Pb concentrations than what we measured (GM = 5.6 µg/L). However, the median Pb concentration in our study (median = 6.3) was higher than has been reported in three previous studies (range of median concentrations in previous studies = 3.8 to 6.0 µg/L) [19,20,22]. Two studies in North Carolina reported blood As concentrations among pregnant women. In the first, the GM blood As concentration (0.45 µg/L) [17] was far lower than reported in our study (1.5 µg/L). The median concentration of As among non-smokers in our study was also more than three times higher than reported for non-smokers in the second North Carolina study (1.5 µg/L vs. 0.40 µg/L) [22]. We are unaware of studies reporting Ni concentrations in blood among pregnant women in the US. A study of Canadian non-pregnant women reported Ni concentrations (GM = 2.2 µg/L) one order of magnitude lower than the concentrations in our study (GM = 26 µg/L) [18]. 

Given the small sample size of this pilot study, our results should be interpreted cautiously. Nonetheless, most other studies of pregnant women living in the U.S. or Canada included predominantly white women with higher levels of education whereas our investigation was restricted to disadvantaged pregnant women in Houston, Texas. The greater Houston area includes a large network of heavily trafficked roadways and both dense (e.g., petrochemical complexes) and dispersed (e.g., metal recycling facilities) industrial activities, which represent potential sources of air emissions of metals (Ni, Cd, As, and Pb). These sources may have contributed to the blood concentrations measured in our study. 

While we did not assess specific exposure sources or routes, the use of biomarkers was preferred as it provides information regarding total personal exposure. Yet, we were only able to collect a single blood specimen during the third trimester. Further, while blood concentrations of the metals examined provide an indication of exposure, blood concentrations of some of the metals may not represent body burden, thus, future studies should measure biomarkers in the matrix most appropriate for the given study question. Additional next steps include examining, among a larger sample of women, changes in metal biomarkers during pregnancy as well as determinants of metals exposure in this population to identify potential points of intervention. 

## 5. Conclusions

We found evidence of exposure to Cd, Pb, Ni, and As among a small group of disadvantaged pregnant women in Houston, Texas. Our study also demonstrates the willingness of this population to participate in several aspects of a future epidemiologic study. Given the susceptibility of pregnant women and infants, and the evidence of adverse maternal and child health effects associated with these compounds [4,5], additional studies to elucidate exposure pathways and explore related health effects experienced by this vulnerable population are well justified. Information obtained in this pilot study will be useful in the design and planning of future health effects studies among this disadvantaged population.

## Figures and Tables

**Table 1:** Demographic characteristics of pregnant women attending a prenatal health clinic serving primarily Medicaid patients.

	All Women (*n* = 31)	Women with a Blood Draw (*n* = 22)
	n (%)	n (%)
Maternal age [mean (SD)]	29.0 (4.9)	30.4 (4.5)
Gestational age at screening [mean (SD)] ^a^	25.3 (4.1)	24.6 (3.8)
Race ^b^		
Black, non-Hispanic	13 (41.9)	9 (40.9)
Hispanic	9 (29.0)	6 (27.3)
White, non-Hispanic	3 (9.7)	3 (13.6)
Other	3 (9.7)	2 (9.1)
Missing	3 (9.7)	2 (9.1)
Marital Status		
Divorced	2 (6.5)	1 (4.5)
In a relationship/married	16 (41.9)	12 (54.5)
Never married	11 (35.5)	8 (36.4)
Missing	2 (6.5)	1 (4.5)
College Degree		
No	23 (74.2)	16 (72.7)
Yes	6 (19.4)	5 (22.7)
Missing	2 (6.5)	1 (4.5)
Annual Household Income		
<$15,000	14 (45.2)	13 (59.1)
$15,000–20,000	5 (16.1)	3 (13.6)
>$20,000	4 (12.9)	4 (18.2)
Don’t know	6 (19.4)	4 (18.2)
Missing	2 (6.5)	1 (4.5)
Smoked during pregnancy (yes)	3 (9.7)	3 (13.6)
Other smokers in the home (yes)	3 (9.7)	2 (9.1)
Works outside home (yes)	11 (35.5)	8 (36.4)
Moved during pregnancy (yes)	6 (19.4)	5 (22.7)

SD, Standard Deviation. ^a^ Eight women were screened and enrolled on different dates, the average days between screening and enrollment for these women was 15.4 days. ^b^ One woman answered white non-Hispanic and Asian and was classified as “other”; 1 woman answered white, non-Hispanic, and Hispanic and was classified as Hispanic.

**Table 2:** Willingness to participate in various future hypothetical study scenarios among 31 pregnant women attending a prenatal health clinic serving primarily Medicaid patients.

In a Future, Hypothetical Research Study of Pregnant Women and Their Babies, Would You Be Willing to….	n	%
allow researchers to obtain a sample of your infant’s cord blood?		
Yes	16	51.6
No	4	12.9
Don’t Know	11	35.5
allow researchers to access to your child’s medical records?		
Yes	14	45.2
No	5	16.1
Don’t Know	12	38.7
allow researchers to access your child’s birth certificate?		
Yes	13	41.9
No	8	25.8
Don’t Know	10	32.3
allow researchers to obtain air samples from inside your home?		
Yes	17	54.8
No	5	16.1
Don’t Know	8	25.8
Refused	1	3.2
allow researchers to obtain air samples from outside your home?		
Yes	27	87.1
No	0	0.0
Don’t Know	3	9.7
Refused	1	3.2
wear a personal badge for 2–3 days?		
Yes	27	87.1
No	2	6.5
Don’t Know	2	6.5
get additional ultrasounds not part of your routine prenatal care?		
Yes	26	83.9
No	2	6.5
Don’t Know	3	9.7
have additional blood draws that are not part of your routine prenatal care?		
Yes	20	64.5
No	7	22.6
Don’t Know	4	12.9

**Table 3:** Distribution of 16 metal compounds in the whole blood (µg/L) of 22 pregnant women attending a primarily Medicaid-serving prenatal clinic in Houston, Texas, 2015–2016.

Metal	LOD	*n* > LOD (%)	GM	Median	Mean ± SD (Range)
Aluminum	0.88	22 (100%)	374	351	581 ± 566 (37.1–2490)
Vanadium	0.07	6 (27%)	0.11	0.05	0.52 ± 1.3 (0.05–5.7)
Chromium	0.09	20 (91%)	1.2	1.7	1.7 ± 0.97 (0.06–3.8)
Manganese	0.09	22 (100%)	5.1	3.7	9.1 ± 11 (0.63–47)
Cobalt	0.09	6 (27%)	0.09	0.06	0.35 ± 0.81 (0.06–3.3)
Nickel	0.10	22 (100%)	26	27	29 ± 12 (11–54)
Copper	0.13	22 (100%)	1744	1821	1838 ± 713 (1059–4546)
Zinc	0.26	22 (100%)	6531	6661	7202 ± 3058 (1488–13,991)
Arsenic	0.14	22 (100%)	1.5	1.4	1.7 ± 0.98 (0.71–5.0)
Selenium	0.48	22 (100%)	77	36	202 ± 276 (17–1011)
Strontium	1.81	20 (91%)	19	33	32 ± 24 (1.3–90)
Silver	0.16	8 (36%)	0.13	0.11	0.22 ± 0.33 (0.11–1.4)
Cadmium	0.21	17 (77%)	0.44	0.57	0.65 ± 0.56 (0.15–2.6)
Antimony	0.10	15 (68%)	3.3	39	34 ± 38 (0.07–146)
Barium	0.20	22 (100%)	767	971	987 ± 658 (84–2582)
Lead	0.21	22 (100%)	5.6	6.3	6.6 ± 4.0 (1.4–20)

LOD, limit of detection; GM, geometric mean.
